# Artificial Intelligence in Medical Education: Transforming Learning and Practice

**DOI:** 10.7759/cureus.80852

**Published:** 2025-03-19

**Authors:** Aadhitya Sriram, Kalpana Ramachandran, Sriram Krishnamoorthy

**Affiliations:** 1 Department of Computer Science, College of Engineering, Guindy Anna University, Chennai, IND; 2 Anatomy, Sri Ramachandra Institute of Higher Education and Research, Chennai, IND; 3 Urology, Sri Ramachandra Institute of Higher Education and Research, Chennai, IND

**Keywords:** ai-assisted assessments, artificial intelligence, medical education, personalized learning, virtual simulations

## Abstract

Artificial intelligence (AI) is reshaping medical education by enhancing learning strategies, improving training efficiency, and offering personalized educational experiences. Traditional teaching methods, such as classroom lectures and clinical apprenticeships, face numerous challenges, including information overload, teaching quality variability, and standardisation difficulties. AI presents innovative, data-driven, and adaptive solutions to overcome these limitations, making medical training more effective and engaging. This study explores how AI can be applied in the field of medical education, also focusing on personalized learning, virtual simulations, assessment methods, and curriculum development. Additionally, it examines the challenges and ethical concerns surrounding AI integration in medical training. A comprehensive literature review was conducted to analyze various AI-driven advancements in medical education. The study delves into adaptive learning platforms, AI-powered simulations, automated assessments, and chatbot-assisted learning. It also reviews recent technological developments to assess AI's impact on medical training and its future potential. AI enhances medical education by tailoring learning experiences to individual student performance, thereby improving knowledge retention and engagement. Virtual simulations and augmented reality provide immersive, hands-on training in a safe environment. Automated assessments facilitate efficient evaluation by offering instant feedback and grading, while AI-driven chatbots assist students in self-directed learning and clinical decision-making. Additionally, AI aids in developing dynamic, data-driven curricula that evolve with the latest medical advancements. However, concerns about data privacy, bias in various AI algorithms, over-reliance on technology, and accessibility disparities must be addressed. AI revolutionises medical education by fostering efficiency, interactivity, and personalized learning. However, its responsible implementation requires addressing ethical and logistical challenges. Future research should focus on ensuring AI fairness, equitable access, and integration with immersive technologies to optimize medical training further.

## Introduction and background

Artificial intelligence (AI) is rapidly revolutionizing medical education by enhancing learning methodologies, improving training efficiency, and providing personalized education experiences [1]. The integration of AI in medical education represents a technological evolution and a paradigm shift in how medical knowledge is acquired, applied, and retained [2].

The traditional model of medical education has been structured around classroom-based learning, cadaver dissections, clinical apprenticeships, and hands-on experience with patients. While these methods have been effective, they come with inherent limitations, including information overload, variability in teaching quality, difficulty in standardizing training across different institutions, and challenges in ensuring personalized learning. AI presents an opportunity to address these limitations by leveraging data-driven approaches, automation, and intelligent feedback mechanisms to create a more efficient and student-centric learning experience [3].

Additionally, AI enables the development of dynamic, adaptive, and engaging interactive learning environments [4]. Traditional textbooks and lectures are often static, requiring students to consume information passively. AI-driven tools such as virtual simulations, interactive case studies, and augmented reality (AR) applications transform learning into an active process where students can engage with real-life scenarios in a risk-free environment [5]. This approach allows hands-on skill development and enhances critical thinking and decision-making abilities.

Another transformative impact of AI in medical education is its ability to provide real-time feedback and assessment. Traditional assessments, such as written exams and oral evaluations, are often time-consuming and may not always reflect a student's competencies [6]. AI-powered assessment tools can analyze performance in real time, providing instant feedback on improvement areas. Machine learning algorithms can evaluate students' diagnostic reasoning, procedural skills, and bedside manner by analyzing their interactions with virtual patients. This immediate and data-driven assessment level allows continuous improvement and ensures that students are adequately prepared for real-world medical practice.

Moreover, AI is critical in bridging the gap between theoretical learning and clinical practice. AI-powered virtual patients and chatbots simulate real-life medical encounters, allowing students to practice history-taking, diagnosis, and treatment planning [7]. These AI-driven tools help students refine their clinical reasoning skills and reduce their dependency on actual patients for early-stage training. This ensures that students gain experience before interacting with live patients.

Beyond student learning, AI also benefits medical educators by streamlining administrative tasks, such as grading, scheduling, and monitoring student progress. AI-driven analytics can help educators identify patterns in student performance, allowing them to adjust teaching strategies accordingly. Furthermore, AI can facilitate curriculum development by analyzing global medical education trends and suggesting updates to ensure the curriculum remains aligned with evolving medical practices. AI-driven simulations enhance traditional hands-on training by providing interactive, risk-free, personalized learning experiences. Unlike cadaver dissections and patient-based learning, AI-based simulations allow students to practice repeatedly, receive instant feedback, and experience adaptive scenarios that evolve based on their responses. Studies indicate virtual simulations improve knowledge retention, diagnostic accuracy, and procedural skills. However, these simulations should complement, not replace, direct patient interactions, as real-world patient care requires empathy, communication, and adaptability - aspects that AI cannot fully replicate.

However, integrating AI into medical education is challenging despite these promising advancements. Ethical considerations must be addressed, such as maintaining patient confidentiality in AI-driven simulations and preventing bias in AI algorithms. There is also the risk of over-reliance on technology, where students may become too dependent on AI-driven tools and neglect developing their independent critical thinking skills. Ensuring equitable access to AI-based learning tools is another challenge, as institutions with limited financial resources may struggle to implement AI-driven solutions effectively. To ensure accessibility in resource-limited settings, we propose developing open-source AI educational tools to reduce costs and promote wider adoption. By leveraging cloud-based AI models, we can minimize hardware requirements, making advanced learning resources available to institutions with limited computing capacity. Creating mobile-friendly AI learning platforms will enable students to access simulations and assessments without expensive equipment. Encouraging government and private sector funding can help subsidize AI-based education tools for underprivileged institutions, further expanding access. Finally, adopting hybrid learning approaches integrating AI-assisted tools with traditional methods will ensure inclusivity and support diverse learning needs.

In the following sections, we will explore the various applications of AI in medical education, its challenges, and the prospects of this transformative technology. AI has the potential to revolutionize medical training, but its implementation must be carefully planned to maximize benefits while mitigating risks. By striking the right balance between technology and traditional medical training, AI can enhance the quality of medical education and prepare future healthcare professionals for an increasingly complex and evolving healthcare landscape.

## Review

Methodology

Search Strategies and Databases

We conducted a comprehensive literature search using multiple databases, including PubMed, Scopus, and Web of Science, to identify relevant studies. The search strategy was developed using a combination of keywords and Boolean operators to ensure a wide yet precise retrieval of literature. The keywords and search queries were predefined and consistently applied across databases.

Inclusion and Exclusion Criteria

Our study selection was guided by well-defined inclusion and exclusion criteria. Studies focusing on AI applications in medical education - such as adaptive learning, virtual simulations, AI-assisted assessments, and curriculum design - were included. We excluded articles that lacked empirical data, were purely theoretical, or did not have direct relevance to medical education. This approach ensured that our findings were based on robust and applicable studies.

Study Selection and Screening Methods

A systematic two-step screening process was followed. First, titles and abstracts were reviewed to identify potentially relevant studies. Subsequently, full-text reviews were conducted to confirm eligibility. Independent reviewers conducted this process to minimize selection bias, with discrepancies resolved through discussion.

Bias Consideration

Although we did not employ formal bias assessment tools, such as the Cochrane Risk of Bias Tool or Newcastle-Ottawa Scale, we critically appraised each study based on factors including study design, sample size, data sources, and the validity of the AI interventions described. Any limitations or potential biases within the included studies were acknowledged in our discussion to ensure a balanced interpretation of findings. These methodological steps were designed to enhance the credibility and reproducibility of our study. 

AI applications in medical education

Personalized Learning and Adaptive Assessments

AI-driven learning platforms, such as intelligent tutoring systems and adaptive learning algorithms, tailor educational content to individual student performance. These platforms dynamically analyze data from student interactions, test performance, and learning behaviours to modify lesson plans and assessments. For instance, AI can identify areas of weakness in a student's knowledge and provide additional resources or quizzes tailored to their specific needs. De et al. assert that social media algorithms offer a thorough and detailed examination of the neurophysiological effects of social media on adolescents, along with the ethical considerations surrounding these impacts [8]. Such systems also support spaced repetition, ensuring students retain information effectively over time.

Moreover, adaptive assessments utilize AI to create customized tests that adjust in real time based on student responses. These assessments accurately measure a student's progress and help educators identify areas requiring further attention. AI enhances student engagement and knowledge retention by offering personalized feedback and targeted learning strategies.

Virtual Simulations and Augmented Reality

AI-powered simulation tools and AR enhance hands-on learning by providing interactive experiences in a risk-free environment. These tools enable medical students and trainees to practice surgical procedures, diagnostic assessments, and emergency response protocols without the need for live patients.

For example, AI-based virtual patients can mimic real-world medical conditions, allowing students to diagnose and treat cases through immersive simulations. These simulations provide real-time feedback and adapt to users' decisions, offering a more comprehensive learning experience. AR-based surgical training systems, such as the use of AI in robotic-assisted surgeries, allow surgeons to practice intricate procedures before performing them in real clinical settings [9,10]. 

**Figure 1:**
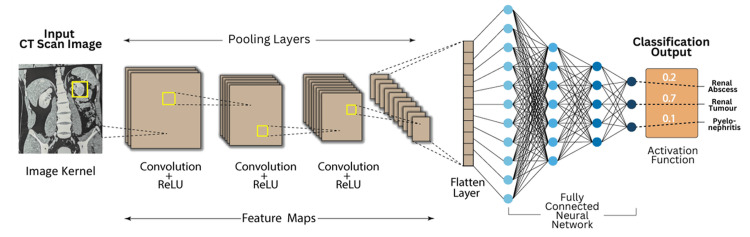
End-to-End Convolutional Neural Network Pipeline for Kidney Disease Identification Reproduced with permission from Rguibi Z et al. [11]. ReLU: Rectified Linear Unit

As demonstrated by Rguibi et al., convolutional neural networks (CNNs) play a crucial role in processing medical imaging data, particularly in the analysis of CT scans. The process begins with the input CT scan, where a region of interest (ROI) is identified, often indicative of potential pathology. As shown in Figure 1, convolutional layers extract essential image features, such as edges, textures, and shapes, by applying filters (also known as kernels) to the input image. The extracted feature maps are then processed through Rectified Linear Unit (ReLU) activation functions, which introduce non-linearity and enhance the network’s ability to capture complex patterns. Following multiple convolutional and pooling layers, the feature maps are flattened and passed through a fully connected neural network to generate a classification output, predicting the likelihood of various renal pathologies, including renal abscess, renal tumour, and pyelonephritis. This structured approach enables CNN models to achieve high accuracy in medical image classification, aligning with the findings reported by Rguibi et al. [11].

The process begins with the input CT scan image, where a region of interest is highlighted by a yellow box, indicating an area with potential pathology. This image transforms convolutional layers. In these layers, convolutional filters (kernels) slide over the image to extract essential features such as edges, shapes and textures. The extracted feature maps are then processed through an activation function, specifically the ReLU, which introduces non-linearity and enables the network to capture complex patterns beyond simple linear transformations.

As the network advances, pooling layers shrink the spatial dimensions of the feature maps while retaining essential information. This down-sampling enhances computational efficiency and mitigates overfitting. The convolutional layers and pooling layers together form a hierarchy of feature extraction, where initial layers detect fundamental image characteristics, and deeper layers identify higher-order features specific to kidney pathologies.

Once feature extraction is complete, the transformed data flows into a fully connected neural network. At this point, the feature maps are flattened into a single vector, enabling the network to process them as structured inputs for decision-making. The fully connected layers establish relationships between extracted features and specific medical conditions, enabling the network to differentiate between various kidney abnormalities.

In the final stage, the output layer applies an activation function, likely a softmax function, to generate probability scores for different possible diagnoses. The output in the image suggests three classification categories: renal abscess, renal tumour, and pyelonephritis, with the model predicting the highest probability for renal tumour (0.7), indicating that it is the most likely diagnosis [12].

Overall, the image illustrates a complete CNN-based diagnostic pipeline for medical imaging. It showcases the transformation of raw CT scan data into a structured classification result, underscoring the importance of convolutional layers for identifying patterns, pooling layers for compressing information, and fully connected layers for generating final predictions. This process highlights the potential of deep learning in enhancing medical diagnostics by enabling automated and highly accurate interpretation of complex imaging data.

Figure 1 illustrates the workflow of imaging-based deep learning applied to kidney diseases, emphasizing its role in diagnosis and classification. The process begins with acquiring CT scan images, followed by CNN layers that extract feature maps through successive convolutions and ReLU activations. Pooling layers refine the feature representation, ultimately feeding into a fully connected neural network that classifies renal conditions such as abscess, tumour, or pyelonephritis. This approach aligns with recent advancements in deep learning for kidney disease imaging, where CNN-based architectures have demonstrated high accuracy in detecting renal abnormalities from medical images. Zhang et al. (2024) emphasized that CNNs have been widely applied in medical imaging for kidney disease diagnosis, particularly in organ segmentation, lesion detection, and tumour classification [13]. Their review highlights the growing role of deep learning in nephrology, demonstrating how automated systems can improve diagnostic accuracy while addressing challenges such as dataset size limitations and model interpretability. In parallel, Zhu et al. (2023) explored deep learning models beyond image-based approaches, utilizing recurrent neural networks (RNNs) with longitudinal electronic health records (EHRs) to predict chronic kidney disease (CKD) progression. Their study showed that temporal models like RNNs could effectively forecast disease advancement from early to late-stage CKD, surpassing traditional machine learning methods [14]. Together, these studies showcase the complementary roles of CNNs in image-based kidney disease detection and RNNs in predictive analytics for disease progression, reinforcing the potential of AI-driven approaches in nephrology.

AI-powered simulators are also used in anatomy education, where students can explore human structures in three-dimensional (3D) models. These models allow students to interact with organs and tissues, improving their understanding of complex anatomical relationships and physiological functions.

AI in Assessment and Feedback

Automated grading systems and AI-based evaluation tools streamline assessments in medical education. Traditional evaluation methods, such as written exams and practical assessments, are time-consuming and subjective. AI-driven solutions help overcome these limitations by providing rapid and objective evaluations. AI-driven grading ensures fairness by applying consistent evaluation criteria, eliminating human subjectivity, and conducting blind assessments based solely on content. It leverages large, diverse datasets to recognize correct responses impartially and uses structured rubrics and NLP for objective evaluation. Additionally, AI systems undergo continuous refinement to minimize biases, and human oversight helps validate complex responses, ensuring a balance between automation and fairness in medical education assessments.

**Figure 2:**
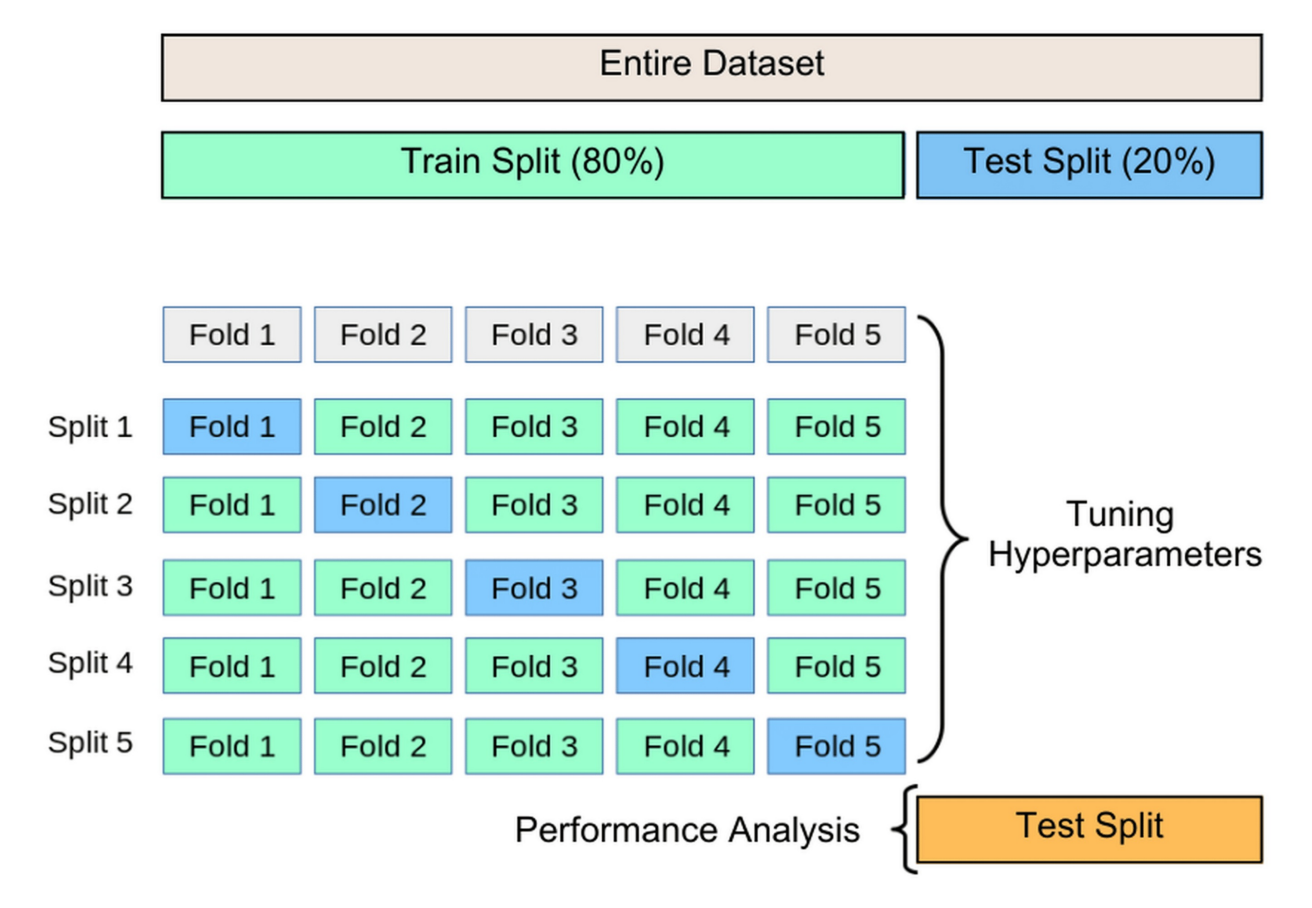
Nested Cross-Validation: Training, Hyperparameter Tuning, and Performance Evaluation Image created by author.

Natural language processing (NLP) algorithms can analyze written responses, providing feedback on the accuracy, clarity, and depth of student explanations. AI-assisted grading of clinical case reports and essays ensure consistency in evaluation while reducing faculty workload. Similarly, AI-driven image recognition tools assess medical imaging interpretations, such as X-rays and MRIs, to determine a student's diagnostic accuracy. In their study on veterinary anatomy, Choudary et al. found that AI-powered diagnostic systems significantly improve clinical examinations, diagnoses, and treatments. These systems enhance accuracy by analyzing vast amounts of data and help veterinarians detect subtle anomalies that might otherwise be overlooked [15]. Nested cross-validation is often employed to ensure robust evaluation of AI-driven assessment tools. Figure 2 illustrates the process of training, validating, and testing AI models using a structured k-fold cross-validation approach.

Figure 2 comprehensively visualizes the k-fold cross-validation process and its role in hyperparameter tuning and model evaluation. It illustrates how a dataset is divided and systematically utilized to ensure the robustness of a machine-learning model before final testing. The above figure illustrates the nested cross-validation approach for training, hyperparameter tuning, and model evaluation, ensuring model robustness before final testing. This systematic validation strategy aligns with the principles of multivoxel pattern analysis (Weaverdyck et al., 2020), which emphasizes structured cross-validation techniques for decoding neural representations in fMRI studies [16]. Both methodologies enhance generalizability and mitigate overfitting by iteratively refining model parameters, making them essential for reliable pattern recognition. Additionally, the consensus features nested cross-validation (cnCV) method (Parvandeh et al., 2020) further refines feature selection by prioritizing stable, informative features across inner folds, reducing overfitting and enhancing model generalizability [17].

The entire dataset is initially split into two significant portions: a training split (80%) and a test split (20%). The training split is used for model development, including feature learning and hyperparameter tuning, while the test split is reserved for final performance evaluation. This ensures the model is assessed on completely unseen data after optimization, providing an unbiased estimate of its generalization capability.

Within the training split, the dataset is divided into five equal-sized folds to facilitate k-fold cross-validation, a widely used resampling technique in machine learning. The dataset is divided into five distinct folds, named Fold 1 through Fold 5. This process ensures a thorough assessment of the model’s performance across various segments of the training data, minimizing overfitting and enhancing its capacity to generalize effectively to unseen data.

During each cycle of cross-validation, a different fold is set aside for validation, while the other four contribute to training. This procedure is carried out five times, guaranteeing that every fold functions as the validation set exactly once. The sequence of training-validation splits is as follows: In the cross-validation process, each split designates a different fold as the validation set while using the remaining folds for training. In Split 1, Fold 1 serves as the validation set, while Folds 2, 3, 4, and 5 are used for training. Similarly, in Split 2, Fold 2 is designated as the validation set, with Folds 1, 3, 4, and 5 contributing to training. In Split 3, Fold 3 is selected for validation, and the remaining folds are used to train the model. In Split 4, Fold four functions as the validation set, while the other folds are utilized for training. Finally, in Split 5, Fold 5 is chosen as the validation set, with the remaining four folds used for training the model.

This iterative validation strategy ensures that the model is evaluated across different data portions, allowing it to be fine-tuned and optimized. The results from these five iterations are averaged to obtain a reliable estimate of the model's performance on validation data. This process aids in hyperparameter tuning, where model parameters, such as learning rates, regularization strengths, and network architectures (in the case of deep learning), are adjusted based on validation performance. Bias in AI-driven assessments can be mitigated by training models on diverse datasets representing various demographics, backgrounds, and learning styles. Implementing bias-detection algorithms helps identify and correct disparities in AI-generated evaluations, while regular human oversight ensures fairness and accuracy. Adaptive assessment models accommodate diverse learning speeds and abilities, thereby avoiding a one-size-fits-all approach. Maintaining transparency in AI decision-making also allows students and educators to understand assessment criteria and challenge potential inaccuracies.

Once cross-validation is complete and the optimal hyperparameters have been determined, the final model is trained using the entire training dataset, excluding the cross-validation dataset. This optimized model is then evaluated using the test split (20%), an independent dataset never seen during training or validation. This ensures that the performance metrics obtained at this stage, such as accuracy, precision, recall, F1 Score, and area under the receiver operating characteristic curve (AUC-ROC), accurately reflect the model's true predictive capability when applied to real-world data. The diagram effectively conveys the structured cross-validation approach, emphasizing the importance of using training data efficiently for both learning and model tuning while keeping a separate test set for unbiased performance evaluation. This methodological framework enhances model robustness by mitigating the risks of overfitting and underfitting, making it a critical step in machine learning model development.

Additionally, AI can track student performance over time, identifying trends and areas that need improvement. Instructors can leverage this information to implement focused interventions, ensuring students gain the support they need to excel academically and thrive in their future clinical roles.

Chatbots and AI-Powered Assistance

AI-driven chatbots and virtual assistants support medical students by answering queries, summarizing complex topics, and guiding clinical decision-making. These tools provide instant access to information, making self-directed learning more efficient. AI-powered assistants, such as IBM Watson and Google Med-PaLM, can analyze vast amounts of medical literature and provide evidence-based recommendations. Students can use these tools to learn about diseases, treatment protocols, and recent research findings. Moreover, AI chatbots integrated into learning management systems (LMSs) can serve as interactive study companions. These chatbots assist students in reviewing material, clarifying doubts, and simulating patient interactions. For instance, AI-powered conversational agents can act as virtual patients, allowing students to practice history-taking, differential diagnosis, and clinical reasoning in a realistic yet controlled setting [18]. AI-driven chatbots have been successfully integrated into medical education to support students in various ways. IBM Watson and Google Med-PaLM assist in answering clinical queries and providing evidence-based recommendations. AI-powered virtual patients simulate real-world scenarios, enabling students to practice history-taking, diagnosis, and treatment planning in a risk-free and interactive environment. Chatbots within LMSs serve as 24/7 virtual tutors, helping students review course material, answer frequently asked questions (FAQs), and reinforce key concepts through quizzes. NLP-based AI assistants provide personalized feedback on written case reports and diagnostic reasoning, enhancing clinical decision-making. By facilitating self-directed learning and reducing faculty workload, these chatbots improve the efficiency and accessibility of medical education.

Data-Driven Curriculum Development

AI helps medical educators refine and optimize curricula by analyzing vast student performance data. Traditional medical education follows a structured curriculum, but AI enables dynamic and evolving syllabi that adapt to the needs of learners.

Machine learning models can analyze student performance metrics, including test scores, practical assessments, and engagement levels, to identify learning gaps. AI can suggest modifications to course content, ensuring that it remains relevant to evolving medical practices and research findings.

Additionally, AI can predict student performance based on learning patterns, allowing educators to provide early interventions for struggling students. Institutions can also utilize AI to track the effectiveness of teaching methods, enabling faculty to make data-driven decisions that improve overall educational outcomes.

Challenges and ethical considerations

Integrating AI into medical education comes with various challenges and ethical considerations that must be carefully managed for responsible deployment. A key issue is safeguarding data privacy and security, as AI depends on extensive datasets that may include confidential student and patient information. Protecting this sensitive data is essential to comply with Health Insurance Portability and Accountability Act (HIPAA) and General Data Protection Regulation (GDPR) regulations. Strong encryption protocols and strict access controls should be enforced to mitigate risks to prevent breaches and unauthorized access to medical records.

Another significant challenge is bias in AI algorithms. If training data is not diverse, AI models may develop biases that affect decision-making. This can lead to disparities in learning experiences and assessments. AI systems must be trained on diverse patient demographics datasets to ensure fair and accurate outcomes. Bias detection mechanisms should be implemented to identify and rectify disparities in AI decision-making, and regular reviews by diverse medical professionals can help improve fairness.

Over-reliance on technology is another concern. While AI enhances medical education, it should complement, not replace, traditional teaching methods and human interactions. Excessive dependence on AI-assisted learning might discourage students from developing essential diagnostic reasoning and decision-making skills. Conventional medical training relies heavily on hands-on experience, peer discussions, and mentorship, and AI-driven platforms should not replace these interactions but enhance them. AI-generated outputs may not always be accurate, so students should be trained to question AI recommendations rather than accepting them unquestioningly. Encouraging problem-solving activities that require human judgment and incorporating hybrid models where AI provides recommendations, but students and faculty validate conclusions can help maintain a balance between AI-driven and human-led training approaches.

Cost and accessibility pose another challenge. Implementing AI-based tools requires significant investment in infrastructure, training, and software. Institutions with limited resources may face difficulties in adopting AI technologies. Wealthier institutions can afford AI advancements, while underfunded medical schools and developing countries may struggle to adopt these technologies. Encouraging government and private sector funding to subsidize AI education tools, promoting open-source AI software to reduce costs, and developing scalable AI solutions that run on low-resource infrastructures, including mobile devices, can help ensure broader access to AI-driven educational tools.

The ethical use of AI in medical training is another important consideration. AI-driven simulations and virtual patients must be designed to accurately reflect real-world medical scenarios while upholding ethical standards. AI should be used responsibly, ensuring it does not replace human judgment in critical decision-making processes. AI-driven virtual patients and simulations should be realistic but not compromise ethics by misrepresenting conditions or oversimplifying complex diagnoses. AI should not replace real-world clinical experiences where empathy, communication skills, and ethical reasoning are critical. Establishing ethical guidelines for AI integration in medical education, ensuring AI models are transparent in their decision-making processes, and using case-based learning where students practice balancing AI input with clinical reasoning and ethical decision-making can help maintain ethical standards [19]. Several strategies can be employed to implement AI-based learning tools in resource-limited institutions effectively. Leveraging open-source AI platforms such as TensorFlow and PyTorch helps reduce costs, while cloud-based AI models minimize the need for high-end hardware and enable remote access to AI-driven resources. Integrating mobile-friendly AI applications enables students to engage with AI-driven learning using their widely available smartphones. Institutions can also seek government and private sector funding to support AI integration in education, advocating for its role as a priority in healthcare training. Additionally, adopting hybrid learning approaches, where AI supplements rather than replaces traditional teaching methods, allows for a gradual and cost-effective transition. By implementing these measures, even resource-limited institutions can benefit from AI-driven advancements in medical education.

AI in medical education presents immense opportunities, but these challenges must be carefully managed. By ensuring data security, minimizing bias, maintaining human oversight, improving accessibility, and promoting ethical usage, AI can significantly enhance medical training while preserving the core values of medical education.

Future directions

The future of AI in medical education is poised to expand significantly as technological advancements evolve. A key advancement lies in merging AI with virtual reality (VR) and AR. This fusion will create deeply immersive and interactive training experiences, providing medical students with realistic practice environments to refine their skills more accurately [20,21]. AI-driven VR and AR simulations will enable learners to engage in realistic clinical scenarios where they can refine their surgical skills, diagnostic abilities, and decision-making processes in a controlled yet dynamic setting. By combining these technologies, students can seamlessly transition from theoretical learning to hands-on experience, equipping them with the necessary skills and confidence before engaging with real patients.

Another significant development is implementing AI-driven personalized medical training. AI algorithms will refine adaptive learning methodologies to tailor educational experiences based on each student's performance, learning pace, and career goals [22]. Through continuous assessment and real-time feedback, AI will help identify individual strengths and weaknesses, allowing customized learning pathways. This level of personalization will ensure that students receive targeted instruction and guidance, optimizing their understanding of medical concepts and improving their overall competency [23].

Future AI-powered simulations will incorporate real-time patient feedback, allowing students to engage with the evolving nature of medical conditions. Unlike static case studies or pre-programmed responses, these simulations will enable learners to witness how diseases progress over time and how treatment approaches impact patient outcomes [24,25]. By interacting with AI-driven virtual patients who display dynamic physiological responses, medical trainees will better understand disease management, patient monitoring, and clinical interventions. This innovation will be particularly beneficial in training students for emergency medicine, intensive care, and chronic disease management.

AI will also play an increasingly vital role in continuous medical education (CME), ensuring that healthcare professionals remain updated with the latest medical advancements. With the rapid evolution of medical knowledge, AI can streamline the process of keeping practitioners informed by providing intelligent content recommendations, curating relevant research articles, and designing performance-based assessments. By analyzing an individual's clinical practice patterns and knowledge gaps, AI-driven CME platforms can suggest tailored learning modules that address specific areas requiring improvement. This will help medical professionals stay at the forefront of their fields and maintain high standards of patient care.

Collaboration between AI and robotics will further enhance medical education, particularly in surgical training [26,27]. AI-powered robotic systems will allow students to practice procedures on AI-controlled robotic patients before performing them on actual patients. These robotic simulators will accurately mimic human anatomy, allowing trainees to refine their motor skills, hand-eye coordination, and precision. AI-driven feedback mechanisms will evaluate student performance in real-time, highlighting areas that need improvement and ensuring that trainees develop the necessary competencies before advancing to live surgical procedures.

Another groundbreaking application of AI in medical education will be using blockchain technology for secure learning records. AI-integrated blockchain systems will provide a tamper-proof method for storing medical credentials, assessments, and certifications. This will simplify the verification process for licensing and credentialing, ensuring that educational achievements and professional qualifications are securely documented and easily accessible [28,29]. Blockchain technology will also facilitate the seamless sharing of electronic records across institutions, enabling medical professionals to transition between educational programs and certifications without the administrative burden of manual verification [30].

By embracing AI-driven innovations, medical institutions can enhance the quality of education and better prepare students for the rapidly evolving healthcare landscape. However, to maximize the potential of AI in medical education, it is essential to ensure that these technologies complement rather than replace traditional training approaches. The human aspects of medical education, such as mentorship, ethical reasoning, and patient interaction, must remain integral to the learning process. AI should serve as an assistive tool that enhances learning outcomes while preserving the core values of medical education. Responsible implementation of AI will ensure that future healthcare professionals are equipped with both the technical expertise and the critical thinking skills necessary to provide high-quality patient care in an increasingly AI-driven healthcare environment.

## Conclusions

Integrating AI in medical education transforms traditional learning methodologies by enhancing interactivity, efficiency, and personalization. AI-driven technologies, including personalized learning pathways, virtual simulations, data-driven curriculum development, and intelligent assessment systems, can significantly improve student engagement and educational outcomes. These advancements provide medical educators with new tools to optimize instructional strategies and offer students adaptive, competency-based learning experiences.

Despite AI's immense benefits to medical education, challenges such as data privacy, algorithmic bias, and disparities in accessibility must be carefully addressed. Ensuring the ethical implementation of AI systems, maintaining transparency in decision-making, and safeguarding the integrity of medical training are paramount. Compliance with regulatory standards, the development of unbiased and representative datasets, and initiatives to improve accessibility will be critical in realizing AI's full potential in medical education.

By adopting AI-driven innovations responsibly and strategically, medical institutions can enhance the quality and effectiveness of medical education. This will better equip students to navigate the complexities of modern healthcare, fostering a future generation of clinicians who are both technologically adept and deeply grounded in patient-centred care.
